# Butterfly eyespot serial homology: enter the Hox genes

**DOI:** 10.1186/1741-7007-9-26

**Published:** 2011-04-28

**Authors:** James Castelli-Gair Hombría

**Affiliations:** 1 CABD, Campus Univ. Pablo de Olavide, Carretera de Utrera km 1, Seville, 41013, Spain

## Abstract

Hox genes modify serial homology patterns in many organisms, exemplified in vertebrates by modification of the axial skeleton and in arthropods by diversification of the body segments. Butterfly wing eyespots also appear in a serial homologous pattern that, in certain species, is subject to local modification. A paper in *EvoDevo *reports the Hox gene *Antp *is the earliest known gene to have eyespot-specific expression; however, not all Lepidoptera express *Antp *in eyespots, suggesting some developmental flexibility.

See research article: http://www.evodevojournal.com/content/2/1/9

## 

A common occurrence during development is the formation of repeated homologous structures. During evolution, some of the elements of such serial homology groups may become specialized to perform specific functions, leading to the morphological diversification of the serial homology elements. The best-studied homology series in animals are the body segments, which initially arise as a series of identical subdivisions and then undergo diversification to give rise to segment-specific structures, such as segments with or without legs in the arthropods or vertebrae with or without ribs in vertebrates. This morphological diversification is regulated by Hox genes. A recent paper published in *EvoDevo *has identified the Antennapedia (*Antp*) Hox gene as the earliest known gene to have eyespot-specific expression [1]. However, *Antp *is not expressed in the eyespots of all Lepidoptera species, uncovering the existence of developmental flexibility in eyespot morphogenesis during evolution.

The pigmented concentric rings observed in eyespots are induced during development by the group of cells located in its centre, called the focus. Nijhout, in early work, discovered that if the focus of the pupal wing is transplanted to another area of the wing, it can induce the formation of an ectopic eyespot in the surrounding cells [2]. Moreover, cauterization of the focus inhibits eyespot formation. These experiments suggested a model (Figure 1A) wherein the focus secretes a signalling molecule, which acts as a morphogen present at decreasing concentration as it spreads away from the source. The cells surrounding the focus would perceive different amounts of morphogen and, depending on the concentration they detect, would activate one or another pigment, giving rise to the pattern of concentric colour rings.

**Figure 1:**
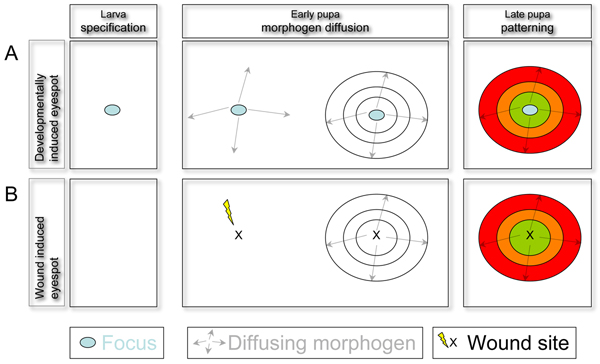
**Schematic representation of normal developmental and wound-induced eyespot formation**. **(A) **The first signs of eyespot specification are observed at the last larval stage with the detection of focus-specific gene expression (light blue circle represents the focus cells). At early pupal stages, signalling molecules are expressed in the focus, from where they spread to neighbouring cells, creating a gradient with higher levels close to the source (arrows represent the diffusing signal). As a response to different concentrations of the signal, the focus and cells surrounding it activate the expression of different transcription factors (represented by different coloured rings around the focus). The area where a transcription factor is expressed depends on the sensitivity of its *cis*-regulatory elements to the morphogen signal activating it and on the possible cross-regulatory interactions between the various transcription factors expressed in the eyespot. These transcription factors will finally activate different pigments generating the adult colour pattern. **(B) **In the absence of larval specification, wound-healing activated signals can induce expression of the transcription factors activating pigmentation. Thus, two distinct pathways can achieve the same morphological outcome, allowing freedom to co-opt different genes while conserving the same final output.

## The morphogen model of eyespot development

The morphogen model has received strong backing over the years since the discovery that several signalling molecules and transcription factors known for their function in controlling *Drosophila *development, in which the morphogen model is well established, are expressed in the focus and in circular patterns in the eyespot of several butterfly species [3-5]. For example, in the Squinting Bush Brown butterfly *Bicyclus anynana*, an important model organism for the study of developmental plasticity and the formation of wing patterning, the focus expresses Wingless (Wg) and phospho-SMAD protein, an indicator of TGFβ signalling activation, suggesting that the focus secretes at least two well-characterized *Drosophila *morphogens [6]. In the Peacock butterfly *Precis coenia *(aka. *Junonia coenia*), there is evidence that the focus expresses high levels of the signalling protein Hedgehog (Hh), which activate its receptor, Patched (Ptc), and the signal transducer Cubitus interruptus (Ci) [3]. Moreover, the Notch (N) receptor is upregulated in the focus of many different butterfly species [1,5]. Besides this richness of signalling pathways, several developmental transcription factors are specifically upregulated in the eyespot, including the Engrailed (En) and the Distalless (Dll) homeodomain proteins, as well as the Spalt (Sal) zinc finger protein [4].

## Temporal regulation of eyespot development

Temporal analysis of gene expression shows that the specification of the eyespot occurs at the last larval stage, when En, Dll, Sal and N become activated in the focus. The recruitment to the eyespot of these developmental genes that are normally required in insects to make the legs and the wings represents a butterfly innovation in the evolution of this novel trait. Later, at the pupal stage, Wg and phospho-SMAD are detected in the focus, followed by the secondary activation of En, Sal and Dll in eyespot cells surrounding the focus [6]. This gives an idea of an early specification of the focus, followed by the activation of a number of ligands that induce around the focus the concentric activation of the transcription factors regulating pigmentation (Figure 1A). Interestingly, in some species, there are many more foci specified at the larval stage than eyespots will later develop, indicating that modulation of the eyespot development cascade at pupal stages may be responsible for the modification of the serial homology elements.

Saenko and collaborators [1] have now discovered that Antp is the earliest transcription factor activated in foci known to date. *Antp *expression appears in all foci primordia, including those that will not contribute to an eyespot in the adult. This indicates that *Antp *function may be important for early eyespot specification, but it is unlikely to be responsible for regulating the departure from the basal serial homology. Thus, in butterflies, *Antp *function has been co-opted to the eyespots, where its function, still to be identified, is unlikely to be the modification of serial homologous structures for which Hox genes are well known.

## Studies of gene function in eyespot support the morphogen model

Although the expression of *Antp *and other developmental genes during eyespot formation is an exciting finding, the field is struggling to obtain functional information about the role each of these genes plays in eyespot formation. This is due to both the sparsity of eyespot mutant variants and the inefficient penetration of RNAi in the butterfly epidermis, where the eyespots form [7]. Current research is aimed at generating new constructs that will help to assay ectopic gene expression and improve RNAi-induced loss of function in butterflies and other organisms [8].

Despite all these difficulties, genetic analysis is slowly confirming some predictions of the morphogen model. Recently, Saenko and collaborators [9] studied three allelic dominant spontaneous mutations (*Bigeye*, *Frodo *and *Spread*) in the *BFS *gene of *Bicyclus anynana*. When heterozygous, these alleles affect eyespot size and colour, and, when homozygous, some allele combinations are lethal, giving rise to embryos with a "segment polarity" phenotype similar to that caused in *Drosophila *by mutations in *en*, *hh *or *wg*. Homozygous *BFS *embryos have normal *wg *and *en *early expression in segmental stripes, but at later embryonic stages, *wg *and *en *are expressed in neighbouring cells, forming wider segmental bands. These experiments indicate that *BFS *controls, directly or indirectly, the maintenance of Wg and Hh signalling in the embryo and probably in the eyespots. Although the *BFS *gene product has not yet been characterized, it is located in a genomic region where there are no conserved genes, suggesting that *BFS *may represent a novel Lepidoptera segment polarity regulator.

## Gene networks in eyespot development

An interesting question is to what extent eyespots are patterned in all butterfly species using the same gene networks. Research in many species has highlighted that eyespots in Nymphalid butterflies and Saturnid moths express *En*, *Sal *and *Dll*, indicating similarities in the way all eyespots are formed. However, recent research shows that there are also many differences. For example, while expression of *En *and *Ci *is preceded in *Junonia coenia *by increased levels of *hh *and *ptc *in the foci [3], in *B. anynana*, *hh *and *ptc *are not upregulated, suggesting that *En *and *Ci *are activated differently in the two species. Similarly, while *Antp *is expressed in the foci of *B. anynana *and five related species, it is not detected in the foci of *Junonia coenia *and two related species [1]. These observations are very intriguing. If, as proposed, eyespots are a novel morphological trait that originated once and was retained and modified in different butterfly species, we may be observing that the developmental gene network that originally gave rise to the eyespot has been modulated differently in various species without modifying the final outcome. This is similar to the different ways in which short and long germ-band insects have modified the early gene network controlling segmentation. Although at the phylotypic stage all insect embryos are morphologically similar, this stage is reached in different ways: in long germ-band insects such as *Drosophila*, the embryo is segmented simultaneously, while in short germ-band insects such as *Tribolium *(the flour beetle), segmentation happens by adding segments sequentially.

How can these developmental differences occur, and where is the developmental plasticity coming from? In the case of the butterfly eyespots, there may be some clues to the possible source of plasticity. It has been observed that injury in the pupal wing induces the formation of ectopic eyespots [2]. At late pupal stages, wound-induced eyespots (Figure 1B) express the same patterning genes as those expressed during normal eyespot development [6], showing that the late eyespot gene network can be deployed correctly using different initial conditions. It may be this kind of flexibility that evolution is exploring to generate the observed developmental gene network diversity.

## Conclusions

Butterfly eyespot development likely conforms to a morphogen model; however, the initiating factors and some downstream components of these genetic pathways may vary among species. Understanding the diversity of eyespot development may shed light on the plasticity of developmental pathways within and between species.
